# Intensive care clinicians’ experiences of palliative withdrawal of mechanical ventilation: a qualitative study

**DOI:** 10.1136/bmjopen-2024-096527

**Published:** 2025-08-08

**Authors:** Nikolaos Efstathiou, Fotini Kristina Michaela Diridis, Michelle Orr, Marianne Baernholdt, Brandi Vanderspank-Wright

**Affiliations:** 1 Department of Nursing and Midwifery, University of Birmingham, Birmingham, UK; 2 Intensive Care Medicine and Anaesthesia, Sandwell and West Birmingham Hospitals NHS Trust, Birmingham, UK; 3 Wright Center for Clinical and Translational Research, VCU Health, Richmond, Virginia, USA; 4 University of Virginia, Charlottesville, Virginia, USA; 5 School of Nursing, University of Ottawa, Ottawa, Ontario, Canada

**Keywords:** Adult intensive & critical care, Adult palliative care, QUALITATIVE RESEARCH

## Abstract

**Abstract:**

**Objectives:**

To explore intensive care unit (ICU) clinicians’ experiences of withdrawing mechanical ventilation during end-of-life care.

**Design:**

An exploratory qualitative design was used, with data collected via semistructured, face-to-face online interviews and analysed using reflexive thematic analysis.

**Participants:**

We recruited ICU clinicians from two hospitals within the West Midlands region of the UK.

**Data collection:**

Semistructured, face-to-face online interviews were used to explore experiences with limitation of life-sustaining treatments in ICU, decision-making and practices for withdrawing mechanical ventilation.

**Findings:**

22 ICU clinicians were interviewed (Physiotherapist=1, Advanced Critical Care Practitioners=4, Physicians=9 and Nurses=8), of which 13 were women (59%). Four themes were developed. (1) Multilayered communication: effective communication was key in planning withdrawal and informing family members, with conflicts arising from cultural differences. (2) Considerations regarding the mode of withdrawing invasive mechanical ventilation: clinicians expressed differing preferences for the method of mechanical ventilation withdrawal. (3) Multiprofessional teamwork: collaborative teamwork was vital, with palliative care practitioners consulted during conflicts or challenging symptoms. (4) Clinicians’ feelings and impact: clinicians empathised with families and experienced psychological burden.

**Conclusions:**

Physician preferences influence the withdrawal process, which is communicated within the multidisciplinary team. Clear protocols can help reduce ambiguity and support less experienced clinicians. Reflection on these practices may help mitigate burnout and compassion fatigue. Further research should examine the effects of physician demographics and patient cultural diversity on the withdrawal process.

**STRENGTHS AND LIMITATIONS OF THIS STUDY**

- By using in-depth interviews, we explored varied experiences and provided new insights into the topic of withdrawing mechanical ventilation from dying patients in the intensive care unit.
- The study was conducted in a region characterised by a high proportion of ethnic minorities and significant socioeconomic challenges, providing an opportunity to explore issues that may not be as prevalent in other areas.
- As this was an exploratory study, findings cannot be generalisable. However, transferability of findings is possible.

## Introduction

 Limiting life-sustaining treatments for critically ill patients in intensive care units (ICUs) is a global practice, often based on patient preferences or the inefficacy of prolonged treatments.^1^ Although practices vary worldwide, most deaths in ICUs follow withholding or withdrawing life-sustaining treatments (WLSTs).^2^

Among the life-sustaining treatments withdrawn, the withdrawal of mechanical ventilation holds a prominent place, with most literature focusing on withdrawing mechanical ventilation as a key component of WLSTs.^3^ There are two primary approaches to withdrawing mechanical ventilation for critically ill patients in an end-of-life context: terminal weaning and terminal extubation. Terminal weaning involves gradually reducing ventilatory support, often resulting in the patient dying with the endotracheal tube in place. Terminal extubation, on the other hand, involves rapidly ceasing mechanical ventilation and removing the tube.^4 5^ Both methods have their advantages and disadvantages. Terminal weaning lowers the risk of upper airway obstruction, reduces dyspnoea, allows for easier analgesia and sedation titration and may be preferred by some families as it offers more time for final interactions.^4^ Terminal extubation prevents prolonged withdrawal, sparing patients, families and staff from undue suffering, and relieves the discomfort of the tube, though it increases the risk of airway obstruction and gasps.^4 5^

Studies across the world report varying rates of extubation during end-of-life care in ICUs, with a systematic review indicating a prevalence between 6% and 83%.^3^ The choice between terminal weaning and extubation is often based on the patient’s consciousness, organ dysfunction and the goal of maintaining patient comfort. Machine learning studies have identified factors influencing the decision to extubate, such as respiratory and vasopressor support levels and respiratory function.^6^

There is a growing body of research on WLSTs, including mechanical ventilation; however, much of it is quantitative in nature, as highlighted in recent systematic reviews.^7 8^ The available qualitative studies have primarily focused on nurses’ experiences, with limited representation of other ICU clinicians, such as physicians, allied health professionals and advanced critical care practitioners (ACCPs).^8^ One qualitative study on ICU clinicians’ experiences of terminal weaning and extubation was conducted in the USA, within a market-driven healthcare system, and before the COVID-19 pandemic^9^; hence, it may not reflect the challenges clinicians currently face in the postpandemic landscape. Given the international variation in end-of-life care practices and the evolving context of ICU care, further qualitative research is needed to explore the experiences of a broader range of ICU clinicians. Such work may help identify ongoing challenges and inform evidence-based approaches to improve end-of-life care for patients and their families.

## Methods

The aim of this study was to explore ICU clinicians’ experiences of withdrawing mechanical ventilation as a part of end-of-life care.

A descriptive qualitative research design was used to address the aim of this study. This design offered the opportunity to provide simple descriptions of participants’ experiences and perceptions, in an area where little is known.^10 11^ In addition, it acknowledged the subjective nature of the problem and the various experiences ICU clinicians have.^11^

### Patient and public involvement

We developed the protocol for this study in consultation with ICU clinicians. Our team included current and past ICU clinicians. Two ICU nurses were consulted while developing the interview guide and refinements were made accordingly.

### Setting and participants

The study took place in two ICUs from two hospitals forming a part of a large National Health Service (NHS) Trust within West Midlands in the UK. The NHS Trust serves areas inhabited by multiple ethnic groups.^12^ The two ICUs have a combined number of 18 beds, and during the study, the ICU staff consisted of 118 nurses, 2 matrons (senior nurses), 14 medical consultants, 13 specialty doctors and 13 ACCPs. There was not a dedicated physiotherapist, but there was daily input by physiotherapists in both ICUs. At the time of the study, both ICUs had a protocol in place to guide care following decisions to withdraw or withhold life-sustaining treatments.

Inclusion criteria for participating in the study included: nurses and allied health professionals should have at least 2 years of ICU experience and physicians at least 1 year of ICU experience (to offer the opportunity for rotating physicians who stay less than 2 years in ICU to participate), be between 21 and 67 years old and work full time. In addition, participants should have the ability to read, understand and speak English. Purposive sampling was used to recruit participants, employing also a maximum variation sampling approach to ensure that a broad experience spectrum of the topic under investigation was captured (eg, junior and senior clinicians, male and female). An ICU consultant acted as a research facilitator in the ICUs, discussing with clinicians the aim of the study and identifying eligible participants. The initial approach was made by the research facilitator, who sent an email to ICU clinicians who met the inclusion criteria. The email included information about the study in the form of a participant information sheet and an informed consent. Those interested in participating responded to the research facilitator, who also collected written informed consents. The details of those who consented were forwarded to the researcher (NE), who contacted them and organised a mutually convenient time for the interviews. As the recruitment was progressing, targeted reminders were sent to specific ICU clinicians who had not responded to the initial invitation to achieve maximum variation in our sample.

### Data collection

In-depth, semistructured, face-to-face online interviews were completed, via Teams (Microsoft), which were audio recorded using an encrypted digital recorder (February 2022–August 2022). Online interviews offered flexibility and accommodated participants’ wishes to be interviewed at a place and time they felt comfortable. A topic guide was developed and used during the interviews, which was based on key issues identified in the relevant literature, discussions with ICU research leads and input from international collaborators (online supplemental file 1). The male interviewer (NE), an experienced qualitative researcher and former ICU nurse, did not have any professional link with the study settings.

### Data analysis

All audio-recorded interviews were transcribed verbatim. Transcripts were reviewed for accuracy and anonymised before analysis. All data were analysed by one researcher (NE). In addition, a sample of the transcripts (n=4) was analysed by a second researcher (FKMD) to ensure a richer interpretation of meaning within our data.^13^ Data were managed on NVivo (v11) and analysed using the interpretative approach of reflexive thematic analysis.^14^ Interview transcripts were read to increase familiarisation with the data, and codes representing the patterns of meaning in the data were initially generated, which were reviewed constantly.^13^ Initial themes were then produced by organising the codes into related and relevant core commonalities, as interpreted by both researchers (NE and FKMD).^15^ In the final stage of analysis, themes were defined and named. Despite the inductive approach used in data analysis, a level of deductive analysis was evident. The deductive component of the analysis was informed by anticipated patterns based on our knowledge of clinical practice and the literature, while the inductive approach allowed the development of new understandings emerging directly from participants’ experiences.

## Findings

### Participants’ characteristics

36 ICU clinicians expressed an interest in participating in the study, of which 28 responded to an invitation to be interviewed. Finally, 22 clinicians were interviewed: one physiotherapist, four ACCPs, nine physicians and eight nurses (table 1). Interviews lasted between 25 and 48 min (average 36 min). The mean age of the participants was 45 years old and had on average 18 years of ICU experience (range 4–33 years). More participants reported being female (13/22), and 14/22 identified as White British. Only three participants reported having special education in palliative care.

**Table 1:** Participants’ characteristics

		No=22 (%)
Gender	Female	13 (59%)
	Male	9 (41%)
Ethnicity	White British/White Other	16 (73%)
	Asian British/Asian	5 (23%)
	African	1 (4%)
Years of ICU experience	≤5	3 (14%)
	6–10	2 (9%)
	11–15	4 (18%)
	16–20	5 (23%)
	≥21	8 (36%)
Education	Bachelor or equivalent degree	9 (41%)
	Postgraduate certificate/diploma	5 (23%)
	MSc	7 (32%)
	PhD	1 (4%)

ICU, intensive care unit.

The 22 interviews allowed us to capture the complex and varied experiences of ICU clinicians and describe fully the target event, indicating saturation.^16^ Following reflexive thematic analysis, four themes were identified: multilayered communication; considerations regarding the mode of withdrawing invasive mechanical ventilation; multiprofessional teamwork and clinicians’ feelings and impact.

### Multilayered communication

Communication was vital during the process of WLSTs, and specifically, the withdrawal of mechanical ventilation. It was evident that communication intensified prior to making a decision to WLSTs and was maintained throughout the process of withdrawal of mechanical ventilation. Communication was multilayered and included multiprofessional communication, communication with the patient, if alert, and more commonly with the family.

For communication to be effective, there was a need for continuity. This was achieved by the nurses acting as links between the patients and their families and the different consultants who rotated between units. Communicating with the family when rapport was already established was also considered important to ensure that information was provided by somebody they already knew. Indeed, communicating difficult news appeared easier when participants perceived that the family was prepared and ready to accept them.

…. We've spoken to the family. I would I always take the nursing staff in with me to talk to the family, to kind of cement their relationship with the family as they care for the patient. You know, and they can answer any questions in my presence and that kind of thing. And that kind of just deepens the rapport. (Participant 9, Medical Consultant).

Healthcare professionals would discuss and decide the most appropriate way to withdraw mechanical ventilation; the plan would be communicated clearly with the nurse looking after the patient. Prior to the withdrawal of mechanical ventilation, healthcare professionals would communicate the process to the family and what to expect.

… you can sort of…pre-empt a lot of potential problems, and maybe someone who’s had some, you know, some difficulty with their airway previously, … good communication with the nurse at the bedside. It’s not that you leave the nurse at the bedside to deal with everything, of course. (Participant 14, ACCP)

So, you speak to the family, prepare the family, discuss with the family of the process that you'll follow and make it clear to them which parameters you are changing. The ventilator will breathe for them, and you explain to the family the ventilator will continue to breathe the patient, but the lack of support that we've removed that is, the oxygen and the inotropes their body will shut down and when that shuts down, we will come and remove the ventilator. (Participant 18, ACCP)

Conflicts were common during communicating views and decisions prior to WLSTs, both between clinicians and clinicians and relatives. Between clinicians, the disagreements tended to be around the timing of transitioning from curative to end-of-life care, which were resolved by communicating the reasoning behind decisions. Conflicts between clinicians and relatives were related to cultural differences or inability to understand that clinicians have in mind the ’best interests’ of the patient.

it’s important to communicate why these decisions are being made so that, especially the junior staff who sometimes might not agree and they still have to carry out the decision… So, it’s important that you convey, and the decision is taken by keeping the whole team in knowledge about what is why we are taking this decision (Participant 17, Medical Consultant).

And I think it’s looked down upon (referring to a faith community) that you didn't do everything that you could have done for that person. You stopped treatment. And so, I think that’s where we get quite a lot of difficult, I don't want to say difficult, but more problematic and incidents within like our work and we get more challenging sort of withdrawals because of the demographical area, the nurses, the teamwork, the communication and then obviously where people’*s training is as well*. (Participant 22, ACCP)

Half of the participants shared experiences of withdrawing mechanical ventilation from ICU patients who were alert and had capacity to understand. In most cases, participants found communicating information about the patient’s deteriorating condition and what would follow distressing, even when the patient was accepting of the inevitable.

He had capacity and he could understand the rational of it. And so I had a chat with him*…* he was of the opinion that he wouldn't want to be on a ventilator if this was just prolonging his life and delaying the inevitable. (Participant 17, Medical Consultant)

### Considerations regarding the mode of withdrawing invasive mechanical ventilation

There were mixed views about the best way to withdraw invasive mechanical ventilation. Some participants were strong supporters of terminal extubation, while others would not consider extubation and always practised only weaning. For those supporting extubation, this was practised after careful planning and prioritising comfort and dignity for the patient. These participants perceived that the endotracheal tube was not allowing for a natural death and was a cause of discomfort for the dying patient.

But leaving the tube in, then it’s not beneficial to the patient and because it’s artificial and you know, death is a natural process, is medicalised in intensive care*…* (extubation) is probably a more natural process. You can see (extubation) as a comfort measure because the tube in itself is uncomfortable and can be distressing, therefore that what you're trying to do is remove as many things that might be distressing for the patient. (Participant 14, ACCP).

Although acknowledged by some participants as a personal preference to extubate, there were some considerations mentioned that were, in most cases, related to the potential of distressing symptoms following extubation, such as respiratory distress and vomiting. Only few of the participants who were supporters of extubation could remember extubation where the patient demonstrated distressing symptoms, which could have an impact on the patient’s relatives or even the whole unit.

… he was extubated with his wife and or child present and for approximately 10 to 15 min… he had an enormous respiratory effort and*…* huge amount of airway noise, uh, which, you know looked awful and sounded dreadful…it was loud throughout the intensive care unit. And although I don't think that he was aware of it cause of his brain injury it looked you know it looked very distressing and that continued until essentially, he died… that’s by far, I think the worst experience that I've had. (Participant 15*,* Medical Consultant).

Some participants did not consider extubation at all during the WLSTs. The act of removing the artificial airway was believed to cause patient discomfort and the immediate death of the patient. It was also believed that weaning was a more ‘kind’ approach for the family present during WLSTs. It was evident that, on occasions, specific professional experiences had shaped these personal views and expectations for patient care. One of the most common alterations on the ventilator during terminal weaning was to reduce the fraction of oxygen to 21% and remove Positive End-Expiratory Pressure (PEEP) and pressure support.

Personally, don't like taking out the ET tube… It feels like you're killing them in such a way so that if I know that they're not, not gonna be able to breathe properly. That’s how it feels to me. And so, I would prefer just to leave the tube in and turn down the support and support them all the way through that. (Participant 13, Nurse)

For most participants, the decision on the mode of withdrawing invasive mechanical ventilation and the process to follow was guided by the patient’s condition and level of respiratory support required at the time of WLST. For example, patients on high fractions of oxygen or with respiratory communicable conditions (eg, COVID-19) would not be considered for extubation. Or patients on high doses of inotropes were expected to die as soon as these were reduced, not giving adequate time to operationalise extubation. For patients with catastrophic brain injury, extubation was more common.

So I think any, any illnesses that might put staff and family members at risk, so patients you know such as the COVID patients or any communicable respiratory illnesses, you know someone’s got PCP (pneumocystis pneumonia) or…COVID maybe in in that scenario you might opt not to just to not try and again expose the environment (Participant 21, Specialty physician).

On any mode of mechanical ventilation withdrawal applied, emphasis was placed on maintaining comfort for the patient by providing adequate analgesia and sedation and supporting the family by providing information and explaining the process and potential symptoms that may be observed while the patient dies.

And I would also ensure that there is the correct medications prescribed, for the removal of ventilation so that the patient doesn't suffer in any way. (Participant 9, Physician Consultant).

…and explained the process of what I was gonna do (to the family present). I'd make sure I've got anticipated meds prescribed and I would prepare them. (Participant 2, Nurse).

### Clinicians’ feelings and impact

Withdrawing mechanical ventilation as a part of the process of WLSTs featured a wide range of feelings. Stress was experienced in association with maintaining care that was at the ‘best interest’ for the patient and morally right. Sometimes, physicians expressed feelings of pressure to consider WLSTs for a patient and, consequently, inform the family. Indeed, these feelings may have led to what was perceived by a small number of participants as avoidance to engage with the withdrawing process.

I'll come in on Monday to start a week having not been on the unit for two or three weeks and nurses at bed spaces will be saying you've got to withdraw this patient…I've got to, you know, assess the patient and find out what’s going on and things…you know, it’s all very well and good when you don't have to make the decision. But when you're the ones who have to make that decision, then stare the family in the eye and tell them this is what the situation is. It’s not an easy decision to make. (Participant 6, Physician Consultant).

I think some individuals avoid this circumstance, because it can be stressful, there can be conflict with the family. So, some clinicians will not engage with this process even though it’s very clear that this is where it’s going. (Participant 9, Physician Consultant)

Participants experienced sadness during the process of withdrawal and occasionally were upset by what seemed like inconsiderate activities in the surrounding ICU environment. Another prominent feeling was that of worrying about how the process is experienced by the family members present. Most participants recognised the impact of the withdrawal process to themselves as well.

I was very, very upset about it, but the rest of the team were there. There was as if nothing happening, you know? I mean like (carrying on with ICU activities). They were not affected by it, so I'm not sure you know (Participant 4, Specialty physician).

I'm always worried about how it looks to, you know, to the family (Participant 11, Nurse).

Formal reflections on withdrawals of mechanical ventilation during regularly held multidisciplinary meetings were described as ways of dealing with feelings and identifying what may have created these feelings and putting measures in place to improve the process. Years of ICU experience was also a mitigating factor in dealing with feelings and emotions.

And then when we do the multi-disciplinary meetings, it’s very nice to look at the results of that (withdrawal of mechanical ventilation) and to say If there’s anything we could have done differently with the actual care. (Participant 13, Nurse).

And I think the longer that I've worked in ICU, the easier it is, I mean it’s not easy and I'm not saying it’s easy but, I think I've developed my own coping mechanisms with this kind of thing now, so I know. And my approach to it, and I kind of know how to box it. (Participant 5, Nurse).

### Multiprofessional teamwork

The withdrawal of mechanical ventilation was planned, organised and operationalised by intensive care physicians and nurses working closely together. Occasionally, palliative care healthcare professionals or chaplains were involved, after being called by ICU clinicians.

Decisions about the operationalisation either of terminal weaning or extubation were collaboratively taken by nurses and physicians, although it was acknowledged that junior staff may not always be confident to partake in these decisions. It was evident throughout the interviews that most ICU clinicians were involved in the operationalisation of withdrawal of mechanical ventilation by being supportive and helping each other during the process.

I think we, you know, we do engage in, in discussion as an MDT (multidisciplinary team) as to how we think this is best to manage. I think as a senior nurse in intensive care, I'm quite comfortable with having that conversation. I think perhaps for certainly more junior staff that their ability to sort of, I suppose challenging and work on experiences is a lot less, isn't it? So, they will very much be guided by what is suggested by the senior, you know, consultant on duty. (Participant 12, Nurse).

I say to them, would you like me to do this? Do you want me to turn the oxygen down? And then, yeah, go from there. And then I've turned to go off and do something else and pop back in 15 more minutes and check that everybody is OK. (Participant 9, Medical Consultant).

Palliative care practitioners were consulted occasionally and were not frequently involved in the care of dying ICU patients. Although some participants felt that they should be involved more, generally the feeling was that these patients are sedated and receive analgesia; hence, as one participant mentioned, *“we're very experienced in managing that and we don't necessarily need them”* (Participant 9, Medical Consultant). However, most of the participants reported that palliative care specialists were consulted on cases when there were conflicts with family members or difficult to control symptoms.

Occasionally we have palliative care involved. I think that’s something that we could most definitely improve on, involving palliative care, so, that the process runs a lot smoother than it sometimes does (Participant 11, Nurse).

…if we've got a family who disagree with the multidisciplinary team’s view that we need to withdraw (mechanical ventilation), then we'll get palliative care involved and they can come and help us talk to the family, … get them involved to help talk to the family because they've got more training in that side. (Participant 6, Medical Consultant).

Despite reports of excellent teamwork, there were examples of ineffective teamwork that created confusion and ambiguity, requiring clinicians to adopt different approaches each time, depending on who they were working with. 17 participants referred to the WLSTs guidance in the units where the study took place, which appeared to reduce ambiguity, improve teamwork and was praised for its easy application in practice.

I think there’s some ambiguity between each nurse and each doctor, to be honest, and I think your relationship with. So certainly, for me, my relationship with each consultant changes, how can, the action that you feel comfortable in taking. (Participant 3, Nurse).

…it (WLSTs guidance) actually forces the clinicians to explicitly state and writing which one of those are and aren't going to be enacted. And so, I think that that’s an approach that I'm comfortable with because it’s forcing people to explicitly make those decisions and justify them and think why and why not it would be appropriate to say. (Participant 20, Specialty physician).

## Discussion

This qualitative study explored ICU clinicians’ experiences of withdrawing mechanical ventilation from critically ill patients transitioning to end-of-life care, following a decision to WLSTs. The mode of mechanical ventilation withdrawal was mostly guided by the patient’s condition, although there were preferences for either terminal weaning or terminal extubation. Multilayered communication was required to ensure that the process of WLSTs and more specifically mechanical ventilation withdrawal was well organised to offer a comfortable and dignified death for the patient. Planning and operationalising the process were mainly undertaken by physicians and nurses, with occasional involvement of palliative care practitioners, particularly when conflicts with family members arose. Local guidelines facilitated the process, reducing confusion and ambiguities. The process inevitably created a variety of feelings and emotions for ICU clinicians, which were ameliorated with experience and a supportive ICU working environment.

Communication between all involved (clinicians, patients that were conscious and family members) was emphasised by all participants in this study as the most important factor in providing good quality end-of-life care. Communication has been identified as one of the quality indicators for end-of-life care in ICUs.^17^ While the previous literature has identified barriers to end-of-life communication in the ICU, such as limited training, attitudes towards death and clinical uncertainty,^17^ our study highlights the impact of physician unit rotation as a further context-specific factor influencing communication dynamics. Few communication-related conflicts were identified between clinicians; however, conflicts with family members were more common, often related to the decision to WLSTs rather than the process itself. Other studies have also reported conflicts with families, frequently attributed to the absence of advance directives, poor communication, a large number of relatives and cultural differences.^18 19^ Cultural differences were the main causes of conflict identified by participants in our study, considering that the setting of this study is in a multicultural region in the UK.

Physicians’ personal preferences influenced the mode of mechanical ventilation withdrawal decision, with preferences shaped by previous experiences and participants’ specialty. Generally, end-of-life decisions vary among ICU clinicians, depending on gender, place of training, specialty and experiences that shape clinicians’ personality and values.^20^ However, most clinicians in this study perceived that decisions were guided by the patient’s condition, discussions with colleagues and family input. Extubation was decided mostly when the patient had less respiratory and vasopressor support, an association also determined by a study using machine learning methods to determine factors associated with the decision to extubate.^6^ Clinicians’ decisions were also influenced by determining the most dignified approach for the patient, and how it would be perceived by the family. Multiple systematic reviews have identified that there is no strong evidence favouring one method over another.^7 21^ Although respiratory distress is more frequent in extubation,^5^ this is manageable and assessment tools and treatment options are available to minimise it.^22^ Although extubation presents a less medicalised death, it may not be preferred by clinicians or families who may favour weaning that provides adequate time to control symptoms and allow families more time with the dying patient.

Participants in this study described experiences where they interacted dynamically with their colleagues, they were working interdependently and adapting to achieve a common goal, all elements of effective teamwork.^23^ Although the ICU multiprofessional team includes a wide range of healthcare professionals, it was evident in the settings where the study took place that only nurses and physicians were actively involved in the palliative withdrawal of mechanical ventilation. In other countries, healthcare professionals, such as respiratory therapists, are also actively involved.^9^ A ‘consultative model’ of palliative care was reported by the ICU clinicians in this study, where palliative care practitioners were consulted mostly when there were conflicts with family members.^24^ It is expected that ICU clinicians possess core palliative care competencies to deal with symptom control during the withdrawal of mechanical ventilation. However, setting goals with a distressed or conflicted family and supporting a grieving family may require insights from a palliative care team to optimise patient care and support the emotional needs of the families involved.^24 25^ Some nurse participants expressed the wish for an ‘integrative model’ of palliative care in the participating ICUs, with more and earlier involvement of palliative care practitioners, a model that has demonstrated positive experiences in terms of improving patient and family care, team dynamics and increasing early formalisation of advance care plans.^26 27^

The existence of a WLST protocol was useful for most participants because it reduced WLST process ambiguity, and it was also offering trigger points to consider for less experienced clinicians. Although the WLSTs process should be individualised, guiding protocols have demonstrated considerable reduction of respiratory discomfort for critically ill patients who were extubated^28 29^ and have been identified as a contributing factor for better quality of end-of-life care.^30^

Despite accepting the inevitability of patients’ conditions that required WLSTs, participants were emotionally affected, with feelings intensified when reflecting on personal experiences and empathising with family members. These feelings can contribute to developing compassion fatigue and burnout syndrome if not addressed. A large UK-based study identified that almost one-thirds of ICU clinicians are at increased risk for burnout syndrome, with female clinicians experiencing higher levels of emotional exhaustion.^31^ Measures were in place within the units where this study took place to reflect formally on WLSTs, and colleagues were supportive of each other.

Participants in this study avoided using the terminology used in most published literature (terminal weaning/extubation), preferring terms, such as palliative or compassionate weaning/extubation. These terms are frequently used in palliative care-focused publications, demonstrating the intent to prioritise 'care' and comfort and allow for natural death to occur.^22 32^

### Limitations

This study took place in a region of the UK with a large proportion of ethnic minorities and with half of households falling within the 20% most socioeconomically challenged areas in England^33^; hence, experiences may have been shaped by this context and may not represent other ICUs covering different populations. However, with the high levels of population movements globally, most countries will experience an influx of ethnic minorities; hence, our findings will be useful and transferable. Although our intention was to interview a wider range of healthcare professionals, it became evident that apart from nurses, ACCPs and physicians, other health professionals were in the periphery in relation to this phenomenon (as we identified following an interview with a physiotherapist). Other professionals, in other ICUs, may have more active involvement and could have offered different insights about the phenomenon. In addition, our sample consisted mostly of participants with more than 6 years of ICU experience. ICU clinicians with less experience in ICU may have different views and experiences. We did not carry out repeat interviews and participants did not provide feedback on the findings; however, the findings were presented during an ICU clinical governance multidisciplinary meeting, where participants had the opportunity to comment on the findings. The small sample size, quite common in qualitative studies, is also a limitation of this study.

## Conclusion

This qualitative study explored the nuanced experiences of ICU clinicians in withdrawing mechanical ventilation as a part of end-of-life care, particularly following decisions to WLSTs. Personal preferences, shaped by experience and specialty, may influence physicians' decisions for either palliative weaning or palliative extubation. While palliative extubation may be favoured in patients with specific conditions, there is still no strong evidence to support palliative extubation over palliative weaning, supporting the notion of a patient-focused approach. Conflicts with family members centred on WLSTs decisions rather than the process itself, with cultural differences being prominent sources of disagreement that need to be explored in future studies.

The importance of multiprofessional teamwork in end-of-life care for ICU patients highlights the need for an integrative model of palliative care. The use of a WLSTs protocol may be beneficial in reducing ambiguity and serve as a valuable guide, especially for less experienced clinicians. Emotional challenges experienced by ICU clinicians necessitate formal reflection processes and supportive environments to mitigate the risk of burnout and compassionate fatigue.

Recommendations for future research include exploring the impact of clinician demographics on WLSTs decisions, evaluating the effectiveness of different communication models and investigating the benefits of an integrative palliative care approach. Additionally, considering the influence of cultural and ethnic diversity on end-of-life decisions is crucial for understanding the broader implications of these findings.

## Supplementary material

10.1136/bmjopen-2024-096527 online supplemental file 1

## Data Availability

Data are available on reasonable request.

## References

[1] Denke C, Jaschinski U, Riessen R (2023). End-of-life practices in 11 German intensive care units. Med Klin Intensivmed Notfmed.

[2] Avidan A, Sprung CL, Schefold JC (2021). Variations in end-of-life practices in intensive care units worldwide (Ethicus-2): a prospective observational study. Lancet Respir Med.

[3] van Beinum A, Hornby L, Ward R (2015). Variations in the Operational Process of Withdrawal of Life-Sustaining Therapy. Crit Care Med.

[4] Paruk F, Kissoon N, Hartog CS (2014). The Durban World Congress Ethics Round Table Conference Report: III. Withdrawing Mechanical ventilation--the approach should be individualized. J Crit Care.

[5] Robert R, Le Gouge A, Kentish-Barnes N (2017). Terminal weaning or immediate extubation for withdrawing mechanical ventilation in critically ill patients (the ARREVE observational study). Intensive Care Med.

[6] Waldauf P, Scales N, Shahin J (2023). Machine learning determination of motivators of terminal extubation during the transition to end-of-life care in intensive care unit. Sci Rep.

[7] Efstathiou N, Vanderspank-Wright B, Vandyk A (2020). Terminal withdrawal of mechanical ventilation in adult intensive care units: A systematic review and narrative synthesis of perceptions, experiences and practices. Palliat Med.

[8] Vanderspank-Wright B, Efstathiou N, Vandyk AD (2018). Critical care nurses’ experiences of withdrawal of treatment: A systematic review of qualitative evidence. Int J Nurs Stud.

[9] Orr S, Efstathiou N, Baernholdt M (2022). ICU Clinicians’ Experiences of Terminal Weaning and Extubation. J Pain Symptom Manage.

[10] Sandelowski M, Barroso J (2003). Classifying the findings in qualitative studies. Qual Health Res.

[11] Doyle L, McCabe C, Keogh B (2020). An overview of the qualitative descriptive design within nursing research. J Res Nurs.

[12] Office for National Statistics (2022). Ethnic group, England and Wales: Census 2021. https://www.ons.gov.uk/releases/ethnicgroupenglandandwalescensus2021.

[13] Byrne D (2022). A worked example of Braun and Clarke’s approach to reflexive thematic analysis. *Qual Quant*.

[14] Braun V, Clarke V (2012). APA handbook of research methods in psychology, Vol 2: research designs: quantitative, qualitative, neuropsychological, and biological.

[15] Braun V, Clarke V (2019). Reflecting on reflexive thematic analysis. Qualitat Res Sport Exerc Health.

[16] Sandelowski M (2008). The SAGE encyclopedia of qualitative research methods.

[17] Visser M, Deliens L, Houttekier D (2014). Physician-related barriers to communication and patient- and family-centred decision-making towards the end of life in intensive care: a systematic review. Crit Care.

[18] Giabicani M, Arditty L, Mamzer M-F (2023). Team-family conflicts over end-of-life decisions in ICU: A survey of French physicians’ beliefs. PLoS One.

[19] Spijkers AS, Akkermans A, Smets EMA (2022). How doctors manage conflicts with families of critically ill patients during conversations about end-of-life decisions in neonatal, pediatric, and adult intensive care. Intensive Care Med.

[20] Nordenskjöld Syrous A, Malmgren J, Odenstedt Hergès H (2021). Reasons for physician-related variability in end-of-life decision-making in intensive care. Acta Anaesthesiol Scand.

[21] Mazzu MA, Campbell ML, Schwartzstein RM (2023). Evidence Guiding Withdrawal of Mechanical Ventilation at the End of Life: A Review. J Pain Symptom Manage.

[22] Ortega-Chen C, Van Buren N, Kwack J (2023). Palliative Extubation: A Discussion of Practices and Considerations. J Pain Symptom Manage.

[23] Ervin JN, Kahn JM, Cohen TR (2018). Teamwork in the intensive care unit. Am Psychol.

[24] Nelson JE, Bassett R, Boss RD (2010). Models for structuring a clinical initiative to enhance palliative care in the intensive care unit: a report from the IPAL-ICU Project (Improving Palliative Care in the ICU). Crit Care Med.

[25] Aslakson RA, Curtis JR, Nelson JE (2014). The Changing Role of Palliative Care in the ICU. Crit Care Med.

[26] Poi CH, Khoo HS, Ang SL (2024). Palliative care integration in the intensive care unit: healthcare professionals’ perspectives – a qualitative study. BMJ Support Palliat Care.

[27] Metaxa V, Anagnostou D, Vlachos S (2021). Palliative care interventions in intensive care unit patients. Intensive Care Med.

[28] Campbell ML, Yarandi HN, Mendez M (2015). A Two-Group Trial of a Terminal Ventilator Withdrawal Algorithm: Pilot Testing. J Palliat Med.

[29] Epker JL, Bakker J, Lingsma HF (2015). An Observational Study on a Protocol for Withdrawal of Life-Sustaining Measures on Two Non-Academic Intensive Care Units in The Netherlands: Few Signs of Distress, No Suffering?. J Pain Symptom Manage.

[30] Mentzelopoulos SD, Chen S, Nates JL (2022). Derivation and performance of an end-of-life practice score aimed at interpreting worldwide treatment-limiting decisions in the critically ill. *Crit Care*.

[31] Vincent L, Brindley PG, Highfield J (2019). Burnout Syndrome in UK Intensive Care Unit staff: Data from all three Burnout Syndrome domains and across professional groups, genders and ages. J Intensive Care Soc.

[32] Lauren FG, Jessica L-S, Jonathan AA, Maureen H (2021). Mechanical ventilation.

[33] West Midlands Combined Authority Equity and Inclusion Scheme 2022-24: Social Inequality.

