# Supplementary material for: Intensive care clinicians’ experiences of palliative withdrawal of mechanical ventilation: a qualitative study
Source: BMJ Open. 2025 Aug 8;15(8):e096527. doi: 10.1136/bmjopen-2024-096527 (PMC12336532; doi:10.1136/bmjopen-2024-096527)
Supplement: online supplemental file 1 [file bmjopen-15-8-s001.docx]

**Supplementary file 1**

**Semi-Structured Interview Guide**

1. Describe your experiences (or an experience) in providing end-of-life care to a ventilated patient in the ICU.
2. Describe your experiences specific to the withdrawal of mechanical ventilation.
3. How do you typically care for patients at the end-of-life who are ventilated? (with respect to discontinuation of mechanical ventilation)

Probes –

- Wean to T-piece, extubate, not extubate?
- Do you wean the support or extubate without weaning?
- If you extubate how do you go about this (walk through process step by step).
- Are there patients at the end of life that you would not extubate? If so, what patient characteristics would lead you to that decisions?
- Who decides on the EOLC plan with respect to the ventilator (ie, MD, nurse, RT, team discussion)?

1. Describe your preference(s) regarding the process of removing ventilator support.
2. What facilitates and/or inhibits the process of withdrawing mechanical ventilation?

Probes –

- Teamwork
- Communication

1. Describe your experiences of discussing ventilator withdrawal with families (and patients if possible).
2. Are you typically comfortable / uncomfortable when there is a decision to extubate a patient as part of their EOLC?  What makes you comfortable / uncomfortable?
3. Have you had a negative experience related to the removal of respiratory support? Please describe further.
