# Supplementary material for: Intensive care clinicians’ experiences of palliative withdrawal of mechanical ventilation: a qualitative study
Source: BMJ Open. 2025 Aug 8;15(8):e096527. doi: 10.1136/bmjopen-2024-096527 (PMC12336532; doi:10.1136/bmjopen-2024-096527)
Supplement: Supplementary file 2 [file bmjopen-2024-096527.reviewer_comments.pdf]

PEER REVIEW HISTORY

BMJ Open publishes all reviews undertaken for accepted manuscripts. Reviewers are asked to complete a checklist review form and are provided with free text boxes to elaborate on their assessment. These free text comments are reproduced below.

ARTICLE DETAILS

Title (Provisional)

Intensive care clinicians' experiences of palliative withdrawal of mechanical ventilation: A qualitative study

Authors

Efstathiou, Nikolaos; Diridis, Fotini Kristina Michaela; Orr, Michelle; Baernholdt, Marianne; Vanderspank-Wright, Brandi

---

VERSION 1 - REVIEW

**Reviewer**	**1**
**Name**	**Fehnel, Corey**
**Affiliation**	**Beth Israel Deaconess Medical Center**
**Date**	**11-Feb-2025**
**COI**	**None**

---

bmjopen-2024-096527

Title: Intensive care clinicians' experiences of palliative withdrawal of mechanical ventilation: A qualitative study

This is important exploratory work for a process with limited high quality evidence to guide practice. Limited description of methodology, and bias from small sample size are major limitations.

Major Comments:

Purposive sampling was referenced as the method used for identifying ICU clinicians but there is very limited detail in how ICU clinicians were selected. What was the operational process used for screening and enrollment of participants in this study? Who were the ICU clinicians that did not participate? Did they differ from ICU clinicians that chose to participate? (e.g. night shift, experience level, et cetera).

What was the topic guide used for interviews? This should be included in supplementary materials submitted with this publication.

Was religious background a theme from interviews? Unless supported by the science I suggest removing references to the Muslim community in the clinican quotations and elsewhere. Perhaps religion as a theme is a barrier in these situations? I don't think any comparisons to other religions was done here, and I do not see any evidence presented that Muslim communities in particular are difficult or challenging.

Related, "the study was conducted in a region characterized by a high proportion of ethnic minorities and significant levels of deprivation, providing an opportunity to explore issues that may not be as prevalent in other areas." Please present the specific demographic data to support this statement. Consider using another phrase to express socioeconomic or other challenges instead of deprivation.

Page 9 line 43. Patients declared dead by neurological criteria have died, and will not exhibit any respiratory effort, or have any potential for distress. While decision making around end of life is germane, discussion of the WMV process for these patients feels out of scope of your primary analysis.

Minor comments:

How was written informed consent obtained if interviews were conducted virtually via Microsoft Teams?

List total N in the title of table 1.

Page 11, line 47, MDT please expound this abbreviation

**Reviewer**	**2**
**Name**	**Schwabe, Sven**
**Affiliation**	**Hannover Medical School, Institute for General Practice and Palliative Care**
**Date**	**12-Jun-2025**
**COI**	**None**

The manuscript presents results of a qualitative study about intensive care clinicians' experiences of palliative withdrawal of mechanical ventilation in two hospitals in UK. Given the increasing number of ventilated patients in intensive care units, this topic is of high clinical relevance. However, the topic has been researched frequently in recent years – including by the lead author (see Orr S, Efstathiou N, Baernholdt M, Vanderspank-Wright B. ICU Clinicians' Experiences of Terminal Weaning and Extubation. J Pain Symptom Manage. 2022 May;63(5):e521-e528. doi: 10.1016/j.jpainsymman.2022.01.016. Epub 2022 Jan 31. PMID: 35093503.) – and the added value of this new qualitative study on the subject is not

entirely clear. If the manuscript will be considered for publication, several methodological and content-related aspects require further elaboration:

Introduction:

Pg 3, Line 3-5:

The research on IUC clinicians' experiences with WLST is summarised in a relatively superficial manner and it is determined that there is a variation in practices of WLST across the countries. This indicates a need for further study to identify the challenges faced by IUC clinicians.... Please elaborate on the specific added value that this study will contribute to the current state of research.

Methods:

Pg 4, Line 1-4:

Why were different inclusion criteria chosen used for a) nurses and allied health professionals (at least two years of ICU experiences) and physicians (at least one year of IUC experience)?

Pg 4, Line 4ff.

A purposive sampling was used to recruit participants, employing also a maximum variation sampling, but in fact, all ICU clinicians were invited to participate in the study? That doesn't make sense to me. Please describe in detail the purposive sampling and the selection of study participants

Data collection:

Which information did the interviewees received in advance about the study? To what extent were the interview data anonymised?

Please add the interview guideline (topic guide) as supplement.

Were all interviews transcribed?

Data analysis:

How did the two researchers cooperate in the qualitative data analysis? Did they analyse different interviews or the same ones? To what extent were the results combined? How were decisions made in cases of differing interpretations?

How exactly were the inductive and deductive coding processes combined? What specifically guided the deductive categories? The interview guide?

Findings:

Pg 11, Multi-professional teamwork

“Decisions about the process were collaboratively taken by nurses and physicians…” is this true? In the “conclusion” (Pg 16,) it is written “physicians’ decision for either palliative weaning or palliative extubation.”

Pg 11, Line 47:

What does “MDT” (interview reference) mean?

Pg 12, line 48:

“…it” – please add, what is meant by “it”

Discussion:

Pg 13, line 35-42:

“In our study, physician unit rotation had an impact on communication, but a systematic review identified additional barriers to EoL ICU communication, including lack of…”

It sounds as if the results of this study contradict the results of the systematic review. In fact, however, barriers were not systematically surveyed in this study.

Pg 13, line 53-56:

“Religious and cultural differences were the main causes of conflict identified by participants in our study, considering that the setting of this study is in a multi-cultural city in UK”

Religious/cultural conflicts were only referred to in this study by means of a single quotation; in my opinion, this statement does not emerge from the study. If religious/cultural conflicts played such a significant role, they should be highlighted in more detail in the results section.

Pg 15, line 15:

“The existence of a WLST protocol was useful for most participants, because it reduced WLST process ambiguity…”

To what extent were appropriate protocols implemented at both stations? Please add this information in the method/setting section.

To what extent were WLST protocols explicitly discussed in the interviews?

Pg 15, line 27-54

The connection between WLST-related stress and job-leaving seems somewhat far-fetched and should be better substantiated.

VERSION 1 - AUTHOR RESPONSE

**Editor’s comments**	**Response to reviewers’ comments**
**Reviewer 1**	

This is important exploratory work for a process with limited high quality evidence to guide practice. Limited description of methodology, and bias from small sample size are major limitations.	Thank you for recognising the importance of this topic. We have considered your concerns about limited description of methodology (as described below), and we have updated the limitations of the study by adding small sample size as a limitation.
Purposive sampling was referenced as the method used for identifying ICU clinicians but there is very limited detail in how ICU clinicians were selected. What was the operational process used for screening and enrollment of participants in this study?<br><br>Who were the ICU clinicians that did not participate? Did they differ from ICU clinicians that chose to participate? (e.g. night shift, experience level, et cetera).	We acknowledge that this information that would explain the purposive sampling approach was missing from the manuscript. The following text was added under Setting and Participants: "The initial approach was made by the research facilitator, who sent an email to ICU clinicians who met the inclusion criteria. The email included information about the study in the form of a participant information sheet and an informed consent. Those interested in participating responded to the research facilitator, who also collected written informed consents. The details of those who consented were forwarded to the researcher (NE), who contacted them and organised a mutually convenient time for the interviews. As the recruitment was progressing, targeted reminders were sent to specific ICU clinicians who had not responded to the initial invitation to achieve maximum variation in our sample."<br><br>Those who did not participate were comparable to those who participated in most characteristics, apart from years of experience. Most participants had more than 6 years of ICU experience. We have recognised this as a limitation of the study, and we have added it under the limitations: "In addition, our sample consisted mostly of participants with more than six years of ICU experience. ICU clinicians with less experience in ICU may have different views and experiences."
What was the topic guide used for interviews? This should be included in supplementary materials submitted with this publication.	A supplementary file of the interview guide has been added to this revised submission.
Was religious background a theme from interviews? Unless supported by the science I suggest removing references to the Muslim community in the clinician quotations and elsewhere. Perhaps religion as a theme is a barrier in these situations? I don't think any comparisons to other religions was done here, and I do not see any evidence presented that	Thank you for this comment.<br>We have removed the reference to the specific faith and any reference to religion. We maintain that there are cultural differences that create conflicts.<br>Quote: And I think it's looked down upon [referring to a faith community] that you didn't do everything that you could have done for that person.

Muslim communities in particular are difficult or challenging.	
Related, “the study was conducted in a region characterized by a high proportion of ethnic minorities and significant levels of deprivation, providing an opportunity to explore issues that may not be as prevalent in other areas.” Please present the specific demographic data to support this statement. Consider using another phrase to express socioeconomic or other challenges instead of deprivation.	Thank you for this comment.<br>Deprivation is a term widely used in the UK (especially with the Indices of Multiple Deprivation); however, we recognise that some readers may not be familiar with this term or its specific indicators.<br>We have added specific demographic data to support the related statement and have used ‘socioeconomic disadvantage’ instead of deprivation: “This study took place in a region of the UK with a large proportion of ethnic minorities and with half of households falling within the 20% most socioeconomic challenged areas in England (35);”
Page 9 line 43. Patients declared dead by neurological criteria have died, and will not exhibit any respiratory effort, or have any potential for distress. While decision making around end of life is germane, discussion of the WMV process for these patients feels out of scope of your primary analysis.	Thank you for this comment. We have removed the “or being neurological dead” from the manuscript.
How was written informed consent obtained if interviews were conducted virtually via Microsoft Teams?	This has been explained and is clearer now in the added content about the recruitment process.
List total N in the title of table 1.	Thank you for identifying this omission. We have added the total N of participants on Table 1.
Page 11, line 47, MDT please expound this abbreviation	We have added an explanation for this abbreviation within the quote [multidisciplinary team]
**Reviewer 2**	
The manuscript presents results of a qualitative study about intensive care clinicians’ experiences of palliative withdrawal of mechanical ventilation in two hospitals in UK. Given the increasing number of ventilated patients in intensive care units, this topic is of high clinical relevance. However, the topic has been researched frequently in recent years – including by the lead author (see Orr S, Efstathiou N, Baernholdt M, Vanderspank-Wright B. ICU Clinicians' Experiences of Terminal Weaning and Extubation. J Pain Symptom Manage. 2022 May;63(5):e521-e528. doi: 10.1016/j.jpainsymman.2022.01.016. Epub 2022 Jan 31. PMID: 35093503.) – and the added value	Thank you for recognising the high clinical relevance of our study.<br>Although there is research available, it tends to be quantitative (as identified in recent systematic reviews by Mazzu et al. Evidence Guiding Withdrawal of Mechanical Ventilation at the End of Life: A Review. J Pain Symptom Manage. 2023 Sep;66(3):e399-e426. doi: 10.1016/j.jpainsymman.2023.05.009; Efstathiou et al. Terminal withdrawal of mechanical ventilation in adult intensive care units: A systematic review and narrative synthesis of perceptions, experiences and practices. Palliat Med. 2020 Oct;34(9):1140-1164. doi: 10.1177/0269216320935002)

of this new qualitative study on the subject is not entirely clear.	The limited qualitative research has focused mainly on nurses' experiences, and there is limited representation of other ICU clinicians in qualitative studies around this topic area. The qualitative study by Orr et al. was conducted in the USA (a different healthcare system from the UK) and prior the COVID-19 pandemic (hence, it does not reflect pos-pandemic challenges).<br><br>We have altered and added content in the last paragraph of the Introduction to enhance the added value of this study.
Introduction:<br>Pg 3, Line 3-5:<br>The research on ICU clinicians' experiences with WLST is summarised in a relatively superficial manner and it is determined that there is a variation in practices of WLST across the countries. This indicates a need for further study to identify the challenges faced by ICU clinicians.... Please elaborate on the specific added value that this study will contribute to the current state of research.	We have altered and added content in the last paragraph of the Introduction to enhance the added value of this study.
Methods:<br>Pg 4, Line 1-4:<br>Why were different inclusion criteria chosen used for a) nurses and allied health professionals (at least two years of ICU experiences) and physicians (at least one year of IUC experience)?	Physicians rotate and may not stay for long in ICU. In order to be inclusive and achieve a maximum variation sample, we set the inclusion criteria to one year for the physicians. This was also one of the recommendations in our consultation with ICU clinicians when we were developing the protocol.<br>We have added an explanation for this in the manuscript: … and physicians at least one year of ICU experience (to offer the opportunity for rotating physicians who stay less than two years in ICU to participate),…
Pg 4, Line 4ff.<br>A purposive sampling was used to recruit participants, employing also a maximum variation sampling, but in fact, all ICU clinicians were invited to participate in the study? That doesn't make sense to me. Please describe in detail the purposive sampling and the selection of study participants.	Thank you for this comment. Additional content has been included demonstrating the recruitment process under Setting and Participants, and how purposive sampling worked. See comment and response to Reviewer 1 on this issue.
Data collection:<br>Which information did the interviewees received in advance about the study?	We hope that the added information under Setting and Participants about the recruitment process answers this question.

To what extent were the interview data anonymised?<br><br>Please add the interview guideline (topic guide) as supplement.<br><br>Were all interviews transcribed?	Transcribed interviews were anonymised before data analysis. We have added this information in the manuscript: “Transcripts were reviewed for accuracy and anonymised before analysis.”<br><br>We have included the interview guide as a Supplementary file.<br><br>We have added in the manuscript under Data analysis the following: “All audio-recorded interviews were transcribed verbatim.”
Data analysis:<br>How did the two researchers cooperate in the qualitative data analysis? Did they analyse different interviews or the same ones?<br><br>To what extent were the results combined?<br><br>How were decisions made in cases of differing interpretations?<br><br>How exactly were the inductive and deductive coding processes combined? What specifically guided the deductive categories? The interview guide?	A sentence was added in the manuscript to indicate that all data was analysed by one researcher and a sample by another researcher. The content of data analysis was reorganised to flow better and have some logic: “All data were analysed by one researcher (NE). In addition, a sample of the transcripts (n=4) was analysed by a second researcher (KD) to ensure a richer interpretation of meaning within our data (12).<br><br>Both analyses were used in the development of the themes. We have altered the content of this section to indicate this.<br><br>The aim was not to achieve consensus but to enhance reflexivity and analytic depth (in line with the reflexive thematic analysis theory). No change in the manuscript.<br><br>We added the following explanation in the manuscript: “The deductive component of the analysis was informed by anticipated patterns based on our knowledge of clinical practice and the literature, while the inductive approach allowed the development of new understandings emerging directly from participants’ experiences. “
Findings:<br>Pg 11, Multi-professional teamwork<br>“Decisions about the process were collaboratively taken by nurses and physicians…” is this true? In the “conclusion” (Pg	Thank you for your comment about this sentence in the Findings, which required clarification.<br>We have corrected the sentence in the findings to: “Decisions about the<br>operationalisation either of terminal weaning

16,) it is written “physicians’ decision for either palliative weaning or palliative extubation.”	or extubation were collaboratively taken by nurses and physicians…”<br>So, physicians decide about the mode of withdrawal, but the operationalisation is a collaborative decision/discussion.
Pg 11, Line 47:<br>What does “MDT” (interview reference) mean?	We have added an explanation for this abbreviation within the quote [multidisciplinary team]
g 12, line 48:<br>“…it” – please add, what is meant by “it”	It is the WLSTs guidance. We have added this explanation in brackets: …it [WLSTs guidance] actually forces the…
Discussion:<br>Pg 13, line 35-42:<br>“In our study, physician unit rotation had an impact on communication, but a systematic review identified additional barriers to EoL ICU communication, including lack of…”<br>It sounds as if the results of this study contradict the results of the systematic review. In fact, however, barriers were not systematically surveyed in this study.	We have rephrased the sentence to clarify the contribution of our study: “While previous literature has identified barriers to end-of-life communication in the ICU, such as limited training, attitudes toward death, and clinical uncertainty (17), our study highlights the impact of physician unit rotation as a further context-specific factor influencing communication dynamics.”
Pg 13, line 53-56:<br>“Religious and cultural differences were the main causes of conflict identified by participants in our study, considering that the setting of this study is in a multi-cultural city in UK”<br>Religious/cultural conflicts were only referred to in this study by means of a single quotation; in my opinion, this statement does not emerge from the study. If religious/cultural conflicts played such a significant role, they should be highlighted in more detail in the results section.	Thank you for this comment.<br>We have removed the reference to the specific faith and any reference to religion. We maintain that there are cultural differences that create conflicts.<br>Quote: *And I think it's looked down upon [referring to a faith community] that you didn't do everything that you could have done for that person.*
Pg 15, line 15:<br>“The existence of a WLST protocol was useful for most participants, because it reduced WLST process ambiguity…”<br>To what extent were appropriate protocols implemented at both stations? Please add this information in the method/setting section.<br><br>To what extent were WLST protocols explicitly discussed in the interviews?	We have added the following information under Setting and Participants: “At the time of the study, both ICUs had a protocol in place to guide care following decisions to withdraw or withhold life-sustaining treatments.”<br><br>There were 30 references to WLST protocols by 17 participants. We have added the number of participants who referred to a WLST protocol in the Findings: “Seventeen participants referred to the WLSTs guidance in the units where the study took place, which appeared to reduce ambiguity, improve teamwork, and was praised for its easy application in practice.”

Pg 15, line 27-54<br>The connection between WLST-related stress and job-leaving seems somewhat far-fetched and should be better substantiated.	Thank you for this comment. We decided to remove this argument. It would require quite a lot of text to make the links clearer. The main points about compassion fatigue and burnout remained.
